# Converse Magnetoelectric Composite Resonator for Sensing Small Magnetic Fields

**DOI:** 10.1038/s41598-019-52657-w

**Published:** 2019-11-08

**Authors:** P. Hayes, M. Jovičević Klug, S. Toxværd, P. Durdaut, V. Schell, A. Teplyuk, D. Burdin, A. Winkler, R. Weser, Y. Fetisov, M. Höft, R. Knöchel, J. McCord, E. Quandt

**Affiliations:** 1 Institute for Materials Science, Kiel University, Kiel, 24143 Germany; 2 Institute of Electrical and Information Engineering, Kiel University, Kiel, 24143 Germany; 3 MIREA - Russian Technological University, Moscow, 119454 Russia; 4 IFW Dresden, SAWLab Saxony, Dresden, 01171 Germany

**Keywords:** Ferroelectrics and multiferroics, Electrical and electronic engineering, Sensors and biosensors

## Abstract

Magnetoelectric (ME) thin film composites consisting of sputtered piezoelectric (PE) and magnetostrictive (MS) layers enable for measurements of magnetic fields passively, i.e. an AC magnetic field directly generates an ME voltage by mechanical coupling of the MS deformation to the PE phase. In order to achieve high field sensitivities a magnetic bias field is necessary to operate at the maximum piezomagnetic coefficient of the MS phase, harnessing mechanical resonances further enhances this direct ME effect size. Despite being able to detect very small AC field amplitudes, exploiting mechanical resonances directly, implies a limitation to available signal bandwidth along with the inherent inability to detect DC or very low frequency magnetic fields. The presented work demonstrates converse ME modulation of thin film Si cantilever composites of mesoscopic dimensions (25 mm × 2.45 mm × 0.35 mm), employing piezoelectric AlN and magnetostrictive FeCoSiB films of 2 µm thickness each. A high frequency mechanical resonance at about 515 kHz leads to strong induced voltages in a surrounding pickup coil with matched self-resonance, leading to field sensitivities up to 64 kV/T. A DC limit of detection of 210 pT/Hz^1/2^ as well as about 70 pT/Hz^1/2^ at 10 Hz, without the need for a magnetic bias field, pave the way towards biomagnetic applications.

## Introduction

Magnetic field sensors are employed in a variety of industrial and electronic device applications, apart from widespread applications like linear position determination or shaft rotation speed sensing (applications where Hall effect and AMR sensors are suited for) there are applications where the magnetic field itself needs to be determined precisely. This holds true for geomagnetic sensing^1^ for mineralogical and navigational purposes^2^, magnetic anomaly detection (MAD)^3^ and remote sensing applications^4^. An emerging field, requiring much higher spatial resolution and compact sensor dimensions, is the contactless imaging or monitoring of biological entities using the magnetic field component of bioelectric currents^5^. This biomagnetic field was unveiled by early studies, proving the concept using giant pickup-coils^6^ later by sensitive yet very complex to operate SQUID magnetometers^7^. Finally, Wikswo^8^ coined the term “Nondestructive testing of humans” more than two decades ago.

The signals emitted from humans in form of magnetic stray fields are of very low amplitude, cardiac signals show amplitudes on the order of 10…100 pT, whereas brain signals are typically one to two orders of magnitude lower^8^. The permanent field of the earth is about six orders of magnitude higher, thus imposing a requirement towards dynamic range. The frequencies of interest range from DC to below 1 kHz^5,8^, which is typically the ELF (extremely low frequency) to VLF (very low frequency) frequency regime.

Many available low-cost, high volume sensor technologies (e.g. Magnetoresistive (xMR) or Hall effect sensors) incrementally improve in performance yet to reach the threshold of being a viable candidate for widespread convenient biomedical sensing operation. Optically pumped magnetometers (OPM) as well as fluxgate magnetometers are promising candidates for the detection of biomagnetic signals. However, despite their room temperature operation and DC field capability they unfortunately exhibit bandwidth and scalability limitations^9^, respectively. In magnetoencephalography (MEG) applications the use of multichannel arrays is anticipated in order to extract useful information^8,10^, thus imposing stringent spatial constraints on any proposed sensor system. xMR^11^ are very promising as they can readily be produced in volume, but yet suffer from excessive 1/f-noise levels^12^. Using flux concentration measures in order to enhance magnetometer sensitivity has proven quite effective^13,14^ but inherently brings a delicate trade-off between sensitivity and spatial resolution.

Continuing efforts of bringing magnetoelectric (ME) devices towards applications^15,16^, especially as sensing elements for most demanding weak fields^17^ in biomagnetic signals are being made^18,19^. By exploiting mechanical resonance enhancement of the direct magnetoelectric effect, the sensitivity can be vastly enhanced for magnetic fields coinciding to the mechanical resonance^20,21^, at the expense of the sensor’s bandwidth. This straightforward approach is completely passive, thus scoring by simplicity, however, low frequency fields are intrinsically tedious to detect. By actively modulating the composite magnetically^22,23^, electrically^24^ or utilizing the delta-E effect^25^ one can up-convert off-resonance signals and thus benefit from resonances and be sensitive in the low frequency regime of interest. Using high frequency surface acoustic wave (SAW) sensors incorporating magnetostrictive material can similarly up convert the magnetic signal by phase modulation^26^.

In this study the converse ME effect in a thin film composite is exploited, exciting a high mechanical resonance mode showing a large vibration amplitude. The signals are detected by a pickup coil which is tuned in order to match its electromagnetic resonance with the excited mechanical resonance of the composite cantilever. Vibrometry measurements give insight to the nature of the mechanical oscillation, leading to periodic magnetisation modulation. The system performance towards small amplitude, low frequency magnetic fields as well as noise behaviour is analysed.

## Methods/Experimental

Thin film ME composites based on silicon are fabricated at Kiel Nanolaboratory using standard microelectromechanical systems (MEMS) cleanroom processes including magnetron sputtering and photolithography. Both active layers, aluminium nitride (AlN) and amorphous iron–cobalt–silicon–boron (FeCoSiB) with a thickness of 2 µm are deposited on adjacent sides of a 350 µm thick double side polished silicon wafer. The FeCoSiB layer is RF magnetron sputtered at 200 W from a 200 mm target (FHR Anlagenbau GmbH, Germany) with a nominal composition of (Fe_90_Co_10_)_78_Si_12_B_10_ at an argon pressure of 6*10^−3^ mbar, using a vonArdenne CS730s cluster sputtering tool. After subsequent depositions of 200 nm material, a pause of 10 minutes allows for cooling and prevents crystallization of the deposited material caused by plasma heating. To promote adhesion to the silicon and prevent ambient oxidation of the alloy, it is sandwiched between thin <10 nm sputtered tantalum layers. The highly textured PE AlN is deposited by reactive magnetron sputtering with nitrogen using a pulse DC source, details are extensively given in^27^. A platinum layer of 80 nm below the AlN serves a dual purpose of seeding the crystal growth as well as to enable electrical contact to the bottom electrode. A top electrode consisting of sputtered chromium (10 nm) and gold (80 nm) functions as a top contact in the PE plate capacitor arrangement. Electrical access to the buried bottom electrode is ensured by partial wet chemical etching of the AlN, for this standard photolithography is used in conjunction with phosphoric acid (H_3_PO_4_) at 80 °C for 20 minutes. The wafers are diced into 25 mm × 2.45 mm dies, which are then heat treated at 270 °C on a hot plate in ambient atmosphere in a magnetic field of 800 Oe directed along the short axis, provided by large permanent magnets. Finally, the silicon dies are bonded to a FR4 PCB board using cyanoacrylate glue thus creating a cantilever structure. The contacts are wire bonded to the carrier PCB. The formed AlN plate capacitor holds a capacity of 1.7 nF off-resonance. Magneto-optical Kerr effect (MOKE) microscopy is performed with a large view polarization sensitive microscope and high power LED illumination, allowing magnetic domain visualization of the magnetic layer. The exact configuration and working principle is described by McCord in^28^. Mechanical characterisation was performed in unshielded environment using a vibrometry system (Polytec, Model UHF-120) and a 5x objective lens, the multicarrier signal in vibration spectroscopy was provided by a vector signal generator (Rhode & Schwarz, Model SMBV100A). The electrical characterization is carried out in a magnetically and electrically shielded environment comprising a multilayer mu-metal cylinder (Aaronia, Model ZG1), further details are given in^29^. The magnetic test field is delivered using a calibrated cylinder coil in conjunction with a low noise AC and DC current source (Keithley, Model 6221), saturation fields are provided by a bi-polar power supply (Kepco, BOP). Analysis of the sensor system, with respect to excitation and readout is performed using a high frequency lock-in amplifier (Zurich Instruments, HF2LI).

## Results and Discussion

**Figure 1:**
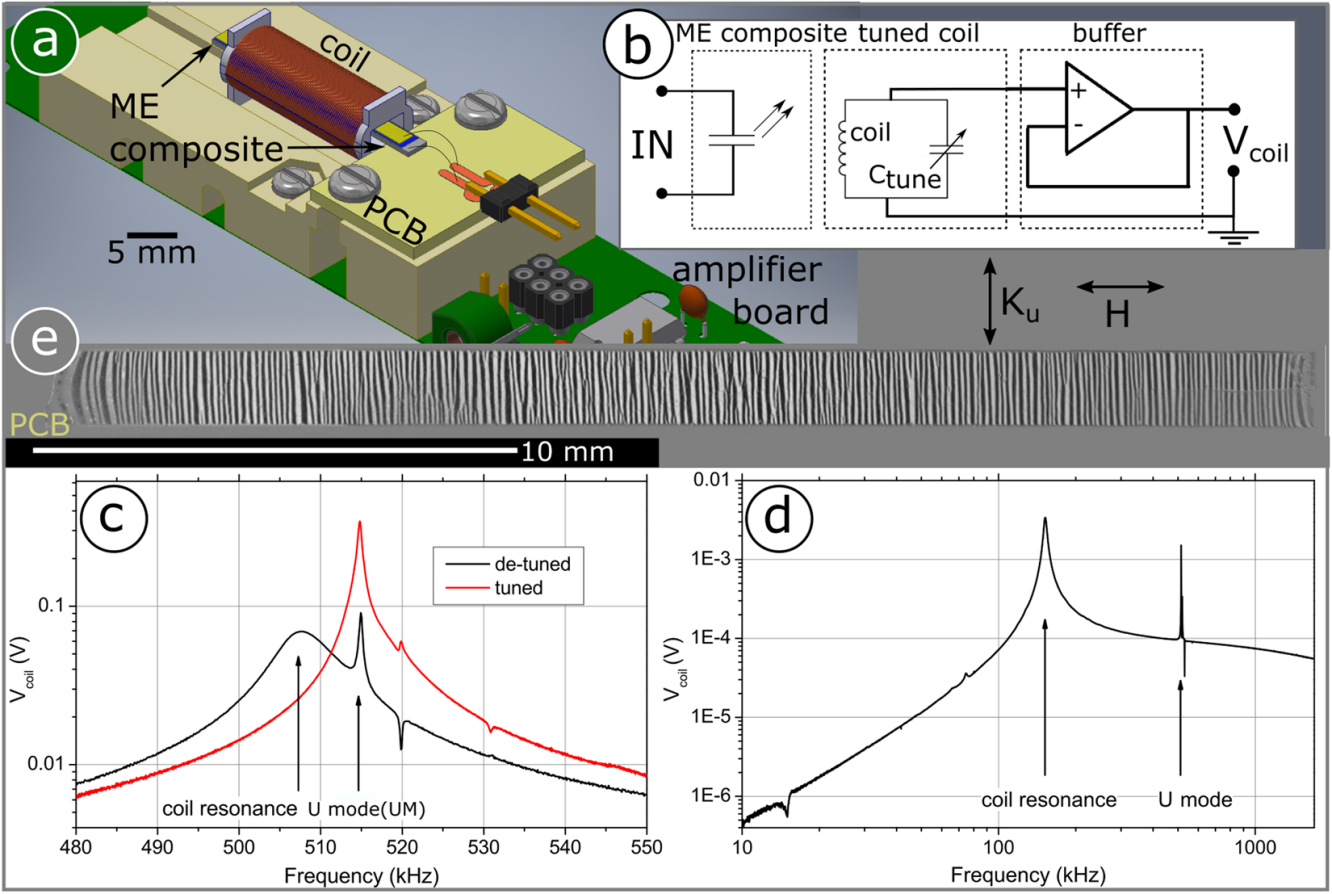
Sensor setup and tuning. (**a**) Schematic representation of the setup mainly consisting of a composite immersed in a pickup coil. The PE plate capacitor forms the input, the tuned pickup coil followed by an amplifier forms the output. (**b**) The circuit depicting the ME composite as a radiative capacitor structure. The signal is induced in the resonant coil, the current is buffered by a low noise unity gain buffer amplifier. (**c**) Frequency response analysis. Pickup coil self-resonance (Q~150) and mechanical resonance frequency (Q~1000), denoted UM. In the de-tuned case, and tuned in order maximize voltage output of the sensor. (**d**) Wide frequency response showing the major effect of the coil resonance and two sharp voltage peaks corresponding to mechanical resonances. (**e**) Large-view MOKE microscopy image showing the full length of the cantilevered composite after magnetic field decay. The magneto optical (MO) domain contrast of the magnetization is directed along the short axis, which is the thermally induced magnetic easy axis, denoted by *K*_u_. *H* indicates the direction of applied magnetic fields, consequently along the magnetically hard axis. The left side is fixed to the PCB.

The fabricated composite is immersed into a coil, which is wound on a polymeric bobbin using 750 windings of 110 µm thick enamelled copper wire. This assembly is in close proximity to a battery powered amplifier board, Fig. 1a. The coil length spawns about 75 percent of the free standing length of the cantilever beam, as is found to be optimum using search coil magnetometers^30^, excluding edge inhomogeneities and local demagnetisation effects of the magnetic core. The copper enclosed area of the coil is 32 mm^2^, the core cross section is 0.005 mm^2^. Using a trimmer capacitor (3…30 pF) in parallel to the coil, its resonance frequency can be tuned downwards, in order to match the excited mechanical resonance. A similar approach has been implemented in fluxgate magnetometers^31,32^, essentially acting as a measure of low noise signal amplification. Figure 1b displays the equivalent circuit schematically. The ME composite forms the input port and is indicated as a radiative capacitor, its high frequency resonance is excited by the internal generator of a lock-in amplifier. The ME composite is inductively coupled to the coil, which is tuned using C_tune_, buffered by a low noise operational amplifier in unity gain configuration (OPA627 of Texas Instruments or LT1128 of now Analog Devices International proved well suited) in order to decouple the resonant circuit from subsequent readout electronics, i.e. the lock-in amplifier. The voltage V_coil_ is fed to the digital lock-in amplifier for synchronous demodulation. Figure 1c shows the system frequency response, denoting the mechanical resonance of interest U mode (UM) at about 515 kHz, which is constant in both traces, whereas the coils self-resonance is about 12 kHz lower in de-tuned and coinciding to UM in the tuned case, leading to increased overall gain. The air coil resonance exhibits equivalent circuit parameters of R_coil_ = 31 Ohm, C_coil_ = 47 pF and L_coil_ = 1.16 mH, which were determined using an Agilent 4294 A assuming a series RLC circuit, using *Q*_coil_ = 1/R_coil_ * (L_coil_/C_coil_)^1/2^ resulting in a coil resonance quality factor of *Q*_coil_ ~ 160. Depending on the state of the magnetic material which is introduced into the coil, the *Q*_coil_ will decrease. The name U mode stems from the fact that a strong U-shaped curvature along the short cantilever axis is formed, as the reader will shortly find out. This U mode mechanical resonance is much sharper, holding a *Q* factor nearly an order of magnitude higher of *Q*_UM_ ~ 1000, tuning does not have to be overly accurate in order to benefit from this resonance convolution. Figure 1d gives a wider view frequency response, revealing only the broad coil self-resonance and two mechanical resonances at about 515 kHz and minor activity at 520 kHz. The resonance mode present at 520 kHz was previously studied under high driving conditions (order of Volts), obtaining a DC resolution of 1.2 nT at 200 mHz^33^.

The U mode proved to be most sensitive to small magnetic fields when excited moderately. Figure 1e shows a MOKE image of the magnetoelastic FeCoSiB thin film on the cantilever, the axis of magnetooptical (MO) sensitivity being vertical. The alternating stripe contrast reveals alternating magnetisation forming due to magnetostatic energy reduction. The orientation of the magnetic domains shows a high degree of orientation along the short cantilever axis, following the thermally induced easy axis of magnetisation, denoted *K*_u_. The observed in-plane stripe-like domains show a rather uniform width of about 60 µm throughout the length of the cantilever. Such a domain structure is typical for thin amorphous films exhibiting low total anisotropy^28^ after magnetic annealing. Externally applied magnetic test fields are directed orthogonally to the sample’s magnetic easy axis, denoted by *H*. In this axis a maximum of magnetoelastic coupling is achieved (Supplemental, Fig. S1). Mechanical clamping is located on the left as indicated. A distortion of the stripe pattern stemming from local magnetoelastic interaction can be seen there, as well as near the free end of the cantilever.

**Figure 2:**
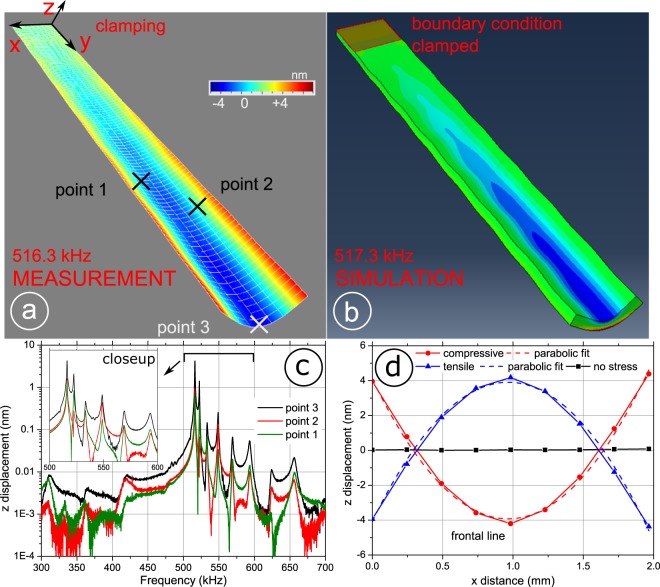
Mechanical oscillation mode analysis. (**a**) Vibrometry measurements of the piezoelectrically excited ME composite, indicating a bending motion along the x (short) axis at a frequency of 514.8 kHz, a high order bending oscillation is superimposed along the *y*-axis. (supplemental video online). (**b**) Qualitative FEM mode analysis simulation of a simple slab sillicon cantilever beam, clamped on one side as indicated, using the physical dimensions of the experiment. Color code gives mises stresses. (**c**) Displacement spectra of the ME composite obtained by multi carrier vibrometry reveals broadband mechanical out-of-plane activity spectra, shown for three different cantilever positions as indicated in a) Inset shows that the mechanical displacement in the U mode is by far domonating. (**d**) Displacement along the *x*-axis at the tip of the cantilever, showing the extremes of one cycle of motion, leading to alternatingly compressive and tensile stress in the magnetostricitve film.

In order to exactly determine the shape of the mechanical U mode of oscillation and gain further insights into the high frequency (out-of-plane) electromechanical spectrum, high speed vibrometry measurements were performed. Figure 2a shows a vibrometry scan of the cantilevers FeCoSiB film surface, piezoelectrically excited at 514.8 kHz with an amplitude of 100 mV. (Supplemental, Vid. S2) The overlay grid indicates the 981 points of measurement; a colour code indicates the out-of-plane displacement relative to the plane of rest. The principal UM oscillation bends symmetrically along the short axis (*x*-axis), this gives rise to the very high resonance frequency of about 515 kHz compared to widely studied flexural modes^33,34^, typically present in the audio frequency regime for mesoscopic silicon structures of millimetre dimensions. The dominant oscillation loss mechanism of flexural modes in long cantilevers lies in air damping^35,36^, thus seldom exceeding a *Q* of few 100 in ambient atmosphere. Due to much less displaced air molecules, this U mode shows inherently less damping, thus leading to a higher value of *Q* than that of low order flexural modes. The maximum displacement along the long *y*-axis amounts to about 25 nm, when excited by 100 mV, thus the resulting curvature contributes marginally to magnetoelastic effects. However, the deflection amplitude towards the free end reaches about 30 nm peak-to-peak displacement along the front most x-line which is about one tenth the cantilevers length, thus giving rise to a vastly increased curvature and accompanying strong magnetoelastic coupling. Note the ripple pattern along this axis, indicating a possibly simultaneously excited high order flexural mode^33^. The frequency of the U mode oscillation deviates by less than 0.7% through a set of five samples, irrespective of the rather large variance introduced by manual die mounting using adhesive glue. The high curvature along the *x*-axis leads to a very pronounced stress induced anisotropy (*K*_***σ***_), uniaxially acting on the magnetostrictive material. This anisotropy is periodically changing its sign, leading to a directionality change of *K*_***σ***_. For tensile stress of the film along the short axis, this results in *K*_***σ***_ being parallel to the thermally induced anisotropy *K*_u_, leading to the addition of these two uniaxial anisotropies. While compressive stress leads to a configuration where *K*_***σ***_ lies along the *y*-axis of the cantilever, thus being orthogonal to *K*_u_, which is quantified using Eq. (1). Where *H*_k_, *µ*_0_ and *M*_s_ are the anisotropy field, the permeability of vacuum and the saturation magnetisation, respectively;

1 $${{\boldsymbol{K}}}_{{\bf{u}}}=\frac{{{\boldsymbol{H}}}_{{\bf{k}}}{{\boldsymbol{\mu }}}_{0}{{\boldsymbol{M}}}_{{\bf{s}}}}{2}$$

*K*_u_ acts along the *x*-axis of the cantilever, for values of *H*_k_ = 880 A/m and *M*_s_= 1.12 MA/m a value of 620 J/m^3^ is obtained.

Figure 2d shows an *x*-axis line scan along the tip of the cantilever, for both extreme cases of oscillation as well as its resting position, along with a parabolic fit matching the shape. Using simple bending analysis after Ohring^37^ Eq. (2) and the parameters gained by the parabolic fit of the measured tip curvature, an estimate of the piezoelectrically induced uniaxial stress (***σ***_*piezo*_) can be made. For the data of an excitation level of 100 mV, a radius of curvature (*R*) along the *x*-axis at the free end of the cantilever of about 8.1 m is obtained, while the substrate to film thickness ratio of *t*_s_ = 350 µm to 2 µm, respectively, makes the film negligibly thin. Therefore following

2 $${{\boldsymbol{\sigma }}}_{{\boldsymbol{piezo}}}=\pm \,\frac{{\boldsymbol{E}}{{\boldsymbol{t}}}_{{\boldsymbol{s}}}}{2{\boldsymbol{R}}}$$

where *E* is the Young’s modulus of 169 GPa, of the Silicon substrate^38^ a peak piezoelectrically induced stress of 3.7 MPa at 100 mV of excitation is estimated. From here, *K*_**σ**_ can be derived, depending on the sign of the piezoelectrically induced stress ***σ***_*piezo*_ and assuming an isotropic magnetostriction (*λ*_*iso*_) of 30 ppm.

3 $${{\boldsymbol{K}}}_{{\boldsymbol{\sigma }}}=\frac{3}{2}{{\boldsymbol{\lambda }}}_{{\boldsymbol{iso}}}{{\boldsymbol{\sigma }}}_{{\boldsymbol{piezo}}}$$

For an excitation amplitude of 100 mV a value for the elastic energy density *K*_***σ***_ of 165 J/m^3^ is generated at the tip. This is one quarter of the value of *K*_u_ meaning the ferromagnetic energy landscape is modulated, yet magnetisation reorientation is not expected. The addition of the two energy densities in the case of tensile stress may lead to domain wall motion already below the energy equilibrium of *K*_***σ***_ and *K*_u_. Exciting the resonance at very high amplitudes in one case even lead to fracture through the entire composite along the centre of the *y*-axis, without facing dielectric breakdown (Supplemental, Fig. S3). Mermelstein *et al*.^39^ studied a magnetoelastic ribbon under stress oscillations and found that the magnetic sensitivity thereof scales with *λ*_iso_/*H*_k_^2^. This ideally demands material having a low anisotropy field, thus a being very soft magnetic, yet magnetostricitive for highest sensitivity.

Vibrational spectroscopy obtained by piezoelectrically exciting multiple frequencies while recording the out-of-plane mechanical activity, on any point of the sample surface, shown in Fig. 2c. The excitation amplitude for these spectral measurements lies below 1 mV per FFT line, because the total power is divided by the number of FFT lines. However, linearity of excitation amplitude with respect to the resulting displacement magnitude is verified up to about 500 mV. The three traces belong to different points on the cantilever surface, as indicated in Fig. 2a. Qualitatively the three spectra show the same principal peaks owing to mechanical resonances, though the excursion amplitudes differ. *Point 1* and *Point 2* are randomly chosen points on the cantilever surface in order to illustrate the validity of the aforementioned. The black trace shows the spectrum belonging to the centre of the tip, denoted *point 3*. The UM resonance mode at 516.3 kHz reveals the highest oscillation amplitude, nearly four times the excursion of the second largest resonance, located at 522.5 kHz. Additional resonances found at 548.8 kHz and 533.4 kHz excurse by at least an order of magnitude lower. Note that this mechanical measurement reveals six clearly distinguishable resonance peaks between 500 and 600 kHz. These resonances are absent in the converse magnetoelectric measurements as shown in Fig. 1d. This may have at least two reasons. First, the magnitude of the stress induced anisotropy is not sufficient to modulate the effective anisotropy, hence only little or no current is induced in the pickup-coil. Second, the mechanical modes are highly symmetric (or to a large extent), leading to effective cancellation of the induced current by nodal points leading to out-of-phase currents within the coil.

A simple mechanical mode analysis was performed using Abaqus CAE 2018 using a uniform mesh size of 50 µm. As both active layers are expected to contribute only negligibly to the overall oscillation behaviour, making up only about 1% of the composite thickness, with no vastly differing Young’s moduli, a simple slab of silicon material having the same geometric dimensions is modelled. In order to best mimic the experimental study, a density of 2.330 g/cm^3^, a Young’s modulus (*E*) of 169 GPa and a Poisson’s ratio (*ν*) of 0.27 was chosen for silicon^38^. Figure 2b shows the modelled resonance mode shape found at a frequency of 517.3 kHz, which matches the experiment to within 0.5%. The asymmetric clamping, on a section of the magnetostrictively coated surface of the structure matches the experiment with a fixed boundary condition. The colour code shows relative stress, indicating maximum compressive strain (deep blue) on the top side and simultaneous tensile strain peaking (red) on the bottom, along the *y*-axis towards the free end (cf. Supplemental, Vid. S2).

**Figure 3:**
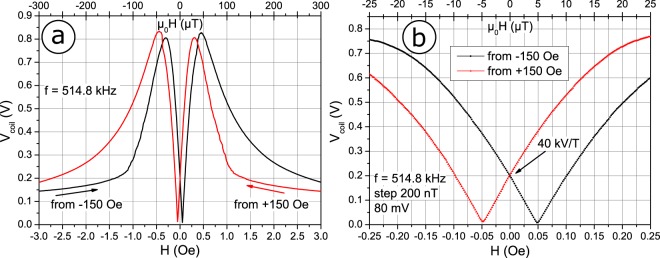
Coil voltage after the buffer amplifier, at the resonant frequency for a drive amplitude of 80 mV with respect to an externally applied field *H*. (**a**) Maximum induced voltage reaches above 800 mV at a field of 0.45 Oe, coming from opposite saturation. At high external fields the induced voltage drops to near zero. Towards zero field the induced amplitude decreases dramatically down to zero at the coercivity. (**b**) Close-up revealing maximum field sensitivity of 40 kV/T at zero bias field. A phase reversal ocurs at the coercive field of 5 µT.

The magnetoelectric sensor provides high dependency of the induced voltage towards low amplitude DC signals, which is presented in Fig. 3. Figure 3a shows the induced RMS coil voltage amplitude after the low-noise amplifier with respect to an externally applied field *H*. When coming from negative saturation the induced voltage reaches above 800 mV at −30 µT, to vastly decrease until it reaches coercivity at 5 µT, where a phase reversal of the induced voltage takes place. This is attributed to the effective magnetic anisotropy switching direction along the *y*-axis of the cantilever, leading to a change of magnetisation and therefore flux direction change within the coil. The obtained loop is highly symmetric with respect to the saturation direction, owing to precise magnetic annealing. At very high magnetic fields, where a saturation of the magnetic material is inevidable, the voltage output again drops to near zero.

**Figure 4:**
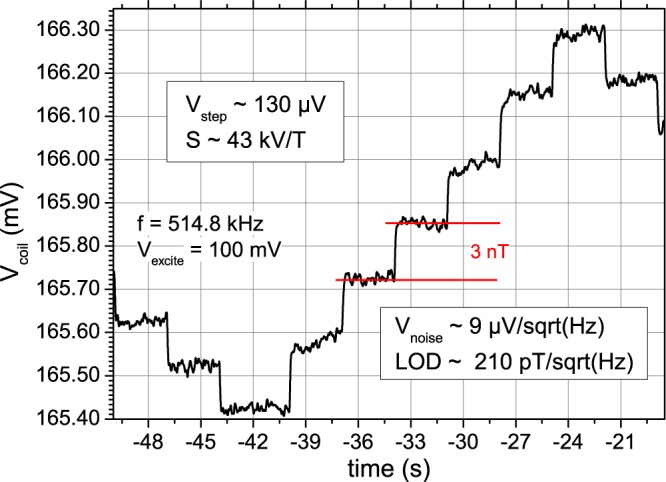
Magnetic staircase signal response, a DC magnetic field step of 3 nT is changed in staircase fashion every 3 seconds. The coil voltage at the modulation frequency changes, giving rise to a DC sensitivity of 43 kV/T. The standard deviation on the steps is about 10 µV, taking into account the noise equivalent bandwidth of the lock-in amplifier, the noise is estimated to be 9 µV/Hz^1/2^.

Figure 4 shows the time-response of the induced coil voltage for a staircase of external DC fields changing every three seconds. The application of small fields relies on the linearity of the transfer function (Fig. 3b) given by the hysteretic behaviour of the amorphous magnetic material, and is well provided for fields through zero. A DC field sensitivity of 43 kV/T is obtained by dividing the output voltage step by the applied magnetic field step of 3 nT, corresponding well to the near-zero slope of Fig. 3b. The standard deviation of the signal on the steps gives rise to the noise floor, by taking into account the equivalent noise bandwidth (ENBW) settings of the lockin amplifier employing a 4^th^ order filter, of about 9 µV/Hz^1/2^, leading to an LOD of about 210 pT/Hz^1/2^ at DC, for a signal-to noise ratio of 1.

From the mechanical (*Q*) and the frequency of vibration (*f*) a bandwidth (*BW*) can be estimated, within which the sensor will be able to detect signals above DC. For a *Q* = 1000 and *f* = 515 kHz, by using *BW* = *f*/(2·*Q*) a low pass characteristic with a −3 dB point of 260 Hz can be estimated^40^. This corner frequency is well within the requirements for most biomagnetic applications.

**Figure 5:**
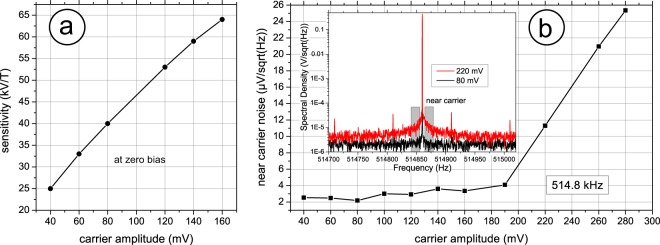
Sensor sensitivity and noise dependence on the applied carrier amplitude. (**a**) The sensitivity at zero field increases nearly linear with increasing carrier voltage (**b**) near carrier noise average, within the low frequency range (<20 Hz) for several carrier amplitudes, two principal regimes are identified, below about 200 mV the noise increases only slightly, roughly doubling over the complete interval, above 200 mV the noise increases nearly sevenfold within 100 mV. (**b**) Inset, shows the noise spectra for 80 mV and 220 mV excitation case, indicating the regime of averaging. At 220 mV of excitation a pronounced pedestal appears as well as broadband noise increase.

The sensitivity towards magnetic fields is given in Fig. 5a, a nearly linear relation with increasing carrier amplitude is found. It can be thought of steepening the slope at zero bias field in Fig. 3b, consequently also leading to increased maximum induced voltages. Unfortunately, this near linear increase in sensitivity is not accompanied by a constant noise floor. Especially in the very low frequency regime of up to 20 Hz, of most interest for biomagnetic sensing, termed near carrier. As Fig. 5b reveals, this near carrier noise is divided into two distinct regimes, for a carrier amplitude below about 200 mV the noise near the carrier essentially doubles from about 2 µV/Hz^1/2^ to 4 µV/Hz^1/2^ for amplitudes ranging from 40 mV to about 200 mV. If the carrier amplitude is further increased, the noise reaches nearly 12 µV/Hz^1/2^ at 220 mV, corresponding to a 20-fold slope compared to the former regime, this increase is by far dominating the benefit of an estimated sensitivity increase to about 85 kV/T at 220 mV. The noise increase in the near carrier regime is more than twofold compared to additional broadband noise (Fig. 5b, inset). This strong noise “pedestal” emerges due to up-conversion of low frequency noise and is connected to periodic magnetization processes within the magnetostrictive phase, leading to such an increase in low frequency noise^41–43^. This directly results in lower detection limits at low frequencies. The broadband noise increase may be attributed to white noise introduced by the emergence of eddy currents generated by excessive magnetization reorientation, initiated by stress anisotropy. Staying below a critical carrier voltage, in this case about 200 mV, will lead to modulation of the effective magnetic anisotropy but not lead to periodic magnetisation sweeping, thus avoiding strong noise contributions connected therewith^44^.

**Figure 6:**
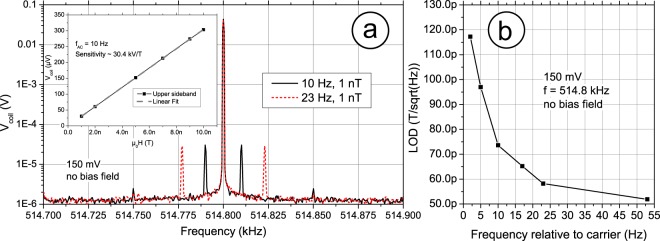
Spectral signal representation taken from behind the pickup coil amplifier. (**a**) Low frequency magnetic signals of 10 Hz and 23 Hz are applied by means of a cylindrical coil at an RMS amplitude of 1 nT. Sidebands form around the carrier due to amplitude modulation. Spurious power line frequency of 50 Hz is also up-converted. No DC magnetic field is present. (**a**) Inset coil voltage indicating the linearity of the modulation with respect to a sinusoidal test signal of 10 Hz at various field amplitudes, using a carrier amplitude of 150 mV. The slope reveals a sensitivity of 30.4 kV/T. (**b**) Limit of detection (LOD) for different test frequencies. An exponential growth in noise towards the carrier limits the performance.

Magnetic fields applied to the excited composite lead to an amplitude modulation at the excitation frequency, Fig. 6a shows typical output spectra. At 514.8 kHz the carrier amplitude is strongly present, corresponding to the U mode mechanical resonance, symmetric sidebands at *f* + *f*_AC_ and *f* − *f*_AC_ correspond to applied AC magnetic fields of 1 nT amplitude and frequencies of 10 Hz (solid) and 23 Hz (dashed). A spurious 50 Hz power line signal is also present symmetric to the carrier. The up-converted spectrum left and right of the carrier, contains the same informational content, hence why amplitude modulation has an inherent efficiency of 50%. The inset shows the linearity towards different AC magnetic field amplitudes. The slope of the fit equals a linear sensitivity of 30.4 kV/T, half of that given in Fig. 5a. Figure 6b shows the limit of detection (LOD) for different AC frequencies, exponentially improving from 117 pT/Hz^1/2^ to 52 pT/Hz^1/2^ at a frequency of 2 Hz to 53 Hz, respectively. The low frequency performance is limited by the up-conversion of 1/f noise.

## Conclusion

A cantilevered mesoscopic ME structure was electrically excited in a mechanical resonance lying in the medium wave regime, at 514.8 kHz exhibiting a mechanical quality factor of about 1000. High speed mechanical vibration spectroscopy reveals that this oscillation leads to large out-of-plane displacements of the structure along its short axis and to high stresses coupled into the magnetostrictive phase. These prove sufficiently strong to alter the magnetic energy landscape by inverse magnetostriction. The observed mechanical U mode is verified by a FEM mode analysis, quantitatively finding a matching resonance frequency at 517.3 kHz, which is within 0.5% of the experimentally determined value. The simulation furthermore suites the experimentally determined deformation pattern.

If placed within a pickup coil the ME oscillator responds strongly to only very few of the determined mechanical vibration modes by sharp induced voltage peaks. The reason for this discrepancy between purely mechanical and converse ME measurements may lie in the fact that a large *K*_***σ***_ is necessary in order to make converse ME interaction effective. Most determined modes lead to weak *K*_***σ***_ and are therefore unable to energetically balance or overcome the statically present induced anisotropy. Furthermore, symmetry of mechanical oscillation modes may lead to spatially localised voltages generated along the structure, which may lie out of phase, effectively cancelling the inductive signal.

If the PE excitation (carrier) signal is set to match the mechanical resonance at 514.8 kHz, its induced voltage amplitude at the excitation frequency is modulated by external magnetic fields. The magnetic field sensitivity increases nearly linearly with carrier signal amplitude, reaching 64 kV/T at 160 mV. Conversely, the noise strongly increases once above an excitation voltage of about 200 mV, leading to excessive stress induced remagnetisation, accompanied domain wall propagation creating a dominant source of noise. Introduction of more sophisticated magnetic layers may lead to restrain of random domain wall motion, i.e. introducing an exchange biased interlayer has proven helpful^45,46^. A magnetic LOD of 210 pT/Hz^1/2^ at DC is determined for a staircase test signal. For a 10 Hz signal an LOD of about 70 pT/Hz^1/2^ was achievable, decreasing noise with distance to the carrier, leads to an LOD of about 50 pT/Hz^1/2^ at 53 Hz. The presented setup enables array integration, as the pickup coil is operated entirely passive and no permanent magnetic bias field is required. Furthermore, a signal bandwidth of DC to 260 Hz meets the criteria of low frequency biomagnetic signals, additional improvement of the LOD is necessary in order to meet demands of biomagnetic signal amplitudes.

## Supplementary information

Supplemental Material

Supplemental 2, Video
