# Supplementary material for: Converse Magnetoelectric Composite Resonator for Sensing Small Magnetic Fields
Source: Sci Rep. 2019 Nov 8;9:16355. doi: 10.1038/s41598-019-52657-w (PMC6841696; doi:10.1038/s41598-019-52657-w)
Supplement: Supplementary file 1 — Supplemental Material [file 41598_2019_52657_MOESM1_ESM.pdf]

Supplemental Material

Converse Magnetoelectric Composite Resonator for Sensing Small Magnetic Fields

P. Hayes[1], M. Jovičević Klug[1], S. Toxværd[2], P. Durdaut[2], V. Schell[1], A. Teplyuk[2], D. Burdin[3], A. Winkler[4], R. Weser[4], Y. Fetisov[3], M. Höft[2], R. Knöchel[2], J. McCord[1] and E. Quandt[1*]

*Correspondence to eq@tf.uni-kiel.de

[1]*Institute for Materials Science, Kiel University, Kiel 24143, Germany*

[2]*Institute of Electrical and Information Engineering, Kiel University, Kiel 24143, Germany*

[3]MIREA - Russian Technological University*, Moscow 119454, Russia*

[4]*IFW Dresden, SAWLab Saxony, Dresden 01171, Germany*

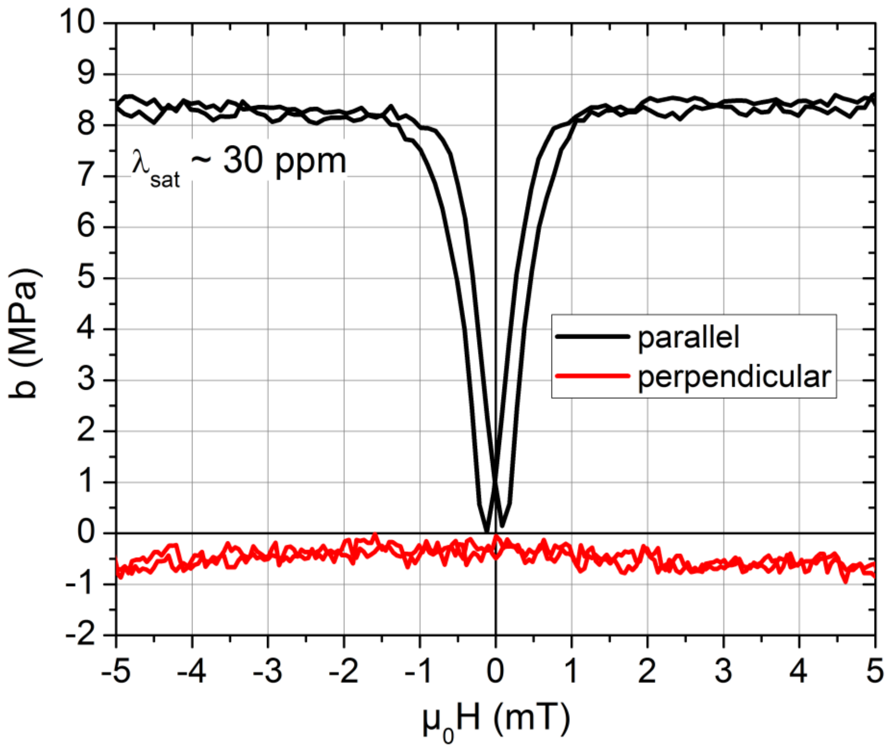

Figure S1 Magnetoelastic measurement of the ME composite, taken by cantilever bending and laser deflection. Magnetic field applied parallel to the long axis (orthogonal to K_U) of the composite yields the highest magnetoelastic response, perpendicular shows nearly no response. In this case the magnetization is already aligned along that axis. Saturation in the parallel case is reached at a field of about 1.4 mT, yielding a saturation magnetostriction coefficient of about 30 ppm.

Supplemental Video S2 Vibrometry measurement depicting the motion of cantilever bending while PE excited at 516.3 kHz in U mode. Every grid crossing corresponds to one measurement point.

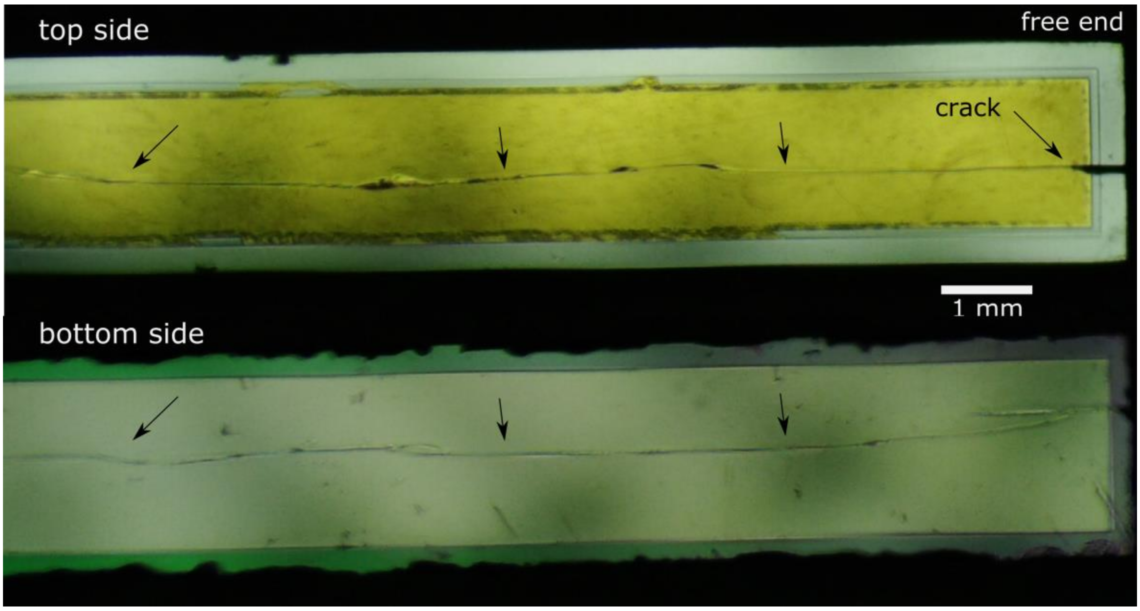

Figure S3 microscopy image of tip region of a fractured ME composite, excited in mechanical resonance at 9 V_{pk}. The piezoelectrically induced fracture lead to mechanical failure though the entire 350 µm thick silicon substrate, as seen by the crack along the center line of the composite. Simultaneous dielectric breakdown of the 2 µm AlN layer did not occur.
